# Periostin Deficiency Increases Bone Damage and Impairs Injury Response to Fatigue Loading in Adult Mice

**DOI:** 10.1371/journal.pone.0078347

**Published:** 2013-10-22

**Authors:** Nicolas Bonnet, Evelyne Gineyts, Patrick Ammann, Simon J. Conway, Patrick Garnero, Serge Ferrari

**Affiliations:** 1 Division of Bone Diseases, Department of Internal Medicine Specialties, Geneva University Hospital, Geneva, Switzerland; 2 INSERM UMR 1033, University of Lyon, Hôpital Edouard Herriot, Lyon, France; 3 Wells Center for Pediatric Research, Indiana University School of Medicine, Indianapolis, Indiana, United States of America; Ohio State University, United States of America

## Abstract

Bone damage removal and callus formation in response to fatigue loading are essential to prevent fractures. Periostin (Postn) is a matricellular protein that mediates adaptive response of cortical bone to loading. Whether and how periostin influences damage and the injury response to fatigue remains unknown. We investigated the skeletal response of *Postn* ^*-/-*^ and *Postn* ^*+/+*^ mice after fatigue stimulus by axial compression of their tibia. In *Postn* ^*+/+*^ mice, cracks number and surface (CsNb, CsS) increased 1h after fatigue, with a decrease in strength compared to non-fatigued tibia. At 15 days, CsNb had started to decline, while CtTV and CtBV increased in fatigued vs non-fatigued tibia, reflecting a woven bone response that was present in 75% of the fatigued bones. Cortical porosity and remodelling also prominently increased in the fatigued tibia of *Postn* ^*+/+*^ mice. At 30 days, paralleling a continuous removal of cortical damage, strength of the fatigued tibia was similar to the non-fatigue tibia. In *Postn* ^*-/-*^ mice, cracks were detectable even in the absence of fatigue, while the amount of collagen crosslinks and tissue hardness was decreased compared to *Postn* ^*+/+*^. Fatigue significantly increased CsNb and CsS in *Postn* ^*-/-*^, but was not associated with changes in CtTV and CtBV, as only 16% of the fatigued bones formed some woven bone. Cortical porosity and remodelling did not increase either after fatigue in *Postn* ^*-/-*^ , and the level of damage remained high even after 30 days. As a result, strength remained compromised in *Postn* ^*-/-*^ mice. Contrary to *Postn* ^*+/+*^ , which osteocytic lacunae showed a change in the degree of anisotropy (DA) after fatigue, Postn^-/-^ showed no DA change. Hence periostin appears to influence bone materials properties, damage accumulation and repair, including local modeling/remodeling processes in response to fatigue. These observations suggest that the level of periostin expression could influence the propensity to fatigue fractures.

## Introduction

Fatigue fractures are due to high repetitive loads and/or alterations of bone microarchitecture and materials properties, favoring microdamage accumulation [1-4]. As a consequence, bone starts to lose its stiffness and strength (toughness) [5,6], and when microcracks eventually reach a critical size, they can result in a fracture [7-9]. Microcracks are normally removed by bone remodeling [10], i.e. through the concerted action of osteoclasts and osteoblasts [11,12]. This process is triggered by osteocyte apoptosis at the site of microdamage, followed by an increased expression of RANKL by adjacent osteocytes and the recruitment of bone resorbing cells [13,14]. 

The bone reaction to fatigue loading involves not only endosteal remodeling, but also a modeling injury response characterized by woven bone formation at the periosteum (callus) [15]. Woven bone is formed when a rapid rate of matrix deposition is needed in order to increase the bone cross sectional area [16], which in turn reduces mechanical strains engendered by loading and contributes to prevent damage accrual [7]. This modeling response depends on the inhibition of sclerostin (Sost) production by osteocytes [17,18]. We reported that down-regulation of Sost expression in response to mechanical loading and parathyroid hormone (PTH) depends on the expression of a matricellular protein, periostin [19,20]. 

The role of periostin in tissue regeneration and repair mechanisms has been previously described in various organs including lung and heart [21]. Besides, periostin expression is increased by inflammation and mechanical stress, suggesting a potential function of this molecule in maintaining the structure and integrity of connective tissues. Periostin expression is also increased at sites of fracture repair [22]. Periostin binds to integrins αvβ3 and αvβ5, regulating cell adhesion and mobility [23,24], and promotes cells survival via the Akt/protein kinase B pathway. Moreover, periostin is linked to type I collagen and thereby may influence bone material properties [19,25,26]. Altogether these observations suggest that periostin could play a role in bone damage and/or in the injury response to fatigue loading.

In order to test this hypothesis, we characterized the skeletal response to fatigue loading in *Postn* ^*-/-*^ mice, i.e. cortical cracks accumulation and repair, as well as callus formation. Here we demonstrate that periostin deficiency alters bone material properties and favors damage accumulation, with delayed remodeling and impaired callus formation. 

## Materials and Methods

### Animals

*Postn Lac Z* knock-in mice (*Postn* ^*-/-*^) were generated as reported previously [27]. *Postn* ^*-/-*^ mice were subsequently bred with C57BL/6J mice, and ear DNA analyzed by PCR was used to identify Postn heterozygous mice. We interbred mice that were heterozygous carriers of this mutation and obtained wild-type (*Postn* ^*+/+*^) and homozygous mutant (*Postn* ^*-/-*^) offspring in the expected Mendelian genetic frequencies. They were subsequently back-crossed for 10 generations, resulting in a genome of 99% C57BL/6J. Mice were housed five per cage, maintained under standard non barriers conditions and had access to water and soft diet ad libitum (Harlan Teklad 2019,SDS, England). Soft diet has been chosen to reduce the malnutrition of the *Postn* ^*-/-*^ mice observed under standard diet due to the enamel and dentin defects of the incisors and molars [27]. 

Three month-old male *Postn* ^*-/-*^ and *Postn* ^*+/+*^ mice were subjected to fatigue loading. After loading the animals received analgesia (I.P 0.05mg/kg buprenorphine). Weight of animal was measured every week. To measure dynamic indices of bone formation, mice received subcutaneous injections of calcein (25mg/kg, Sigma, Buchs, Switzerland) 9 and 2 days before euthanasia. At 1h, 3 days, 15 days or 30 days after fatigue the animals were killed and blood was collected for serum measurements. Tibias were excised for micro-computed tomography analysis, histomorphometry, microcracks evaluation, macro and nano-biomechanical analysis and gene expression. Animal procedures were approved by the University Of Geneva School Of Medicine Ethical Committee and the State of Geneva Veterinarian Office.

### In vivo mechanical loading

**Figure 1:**
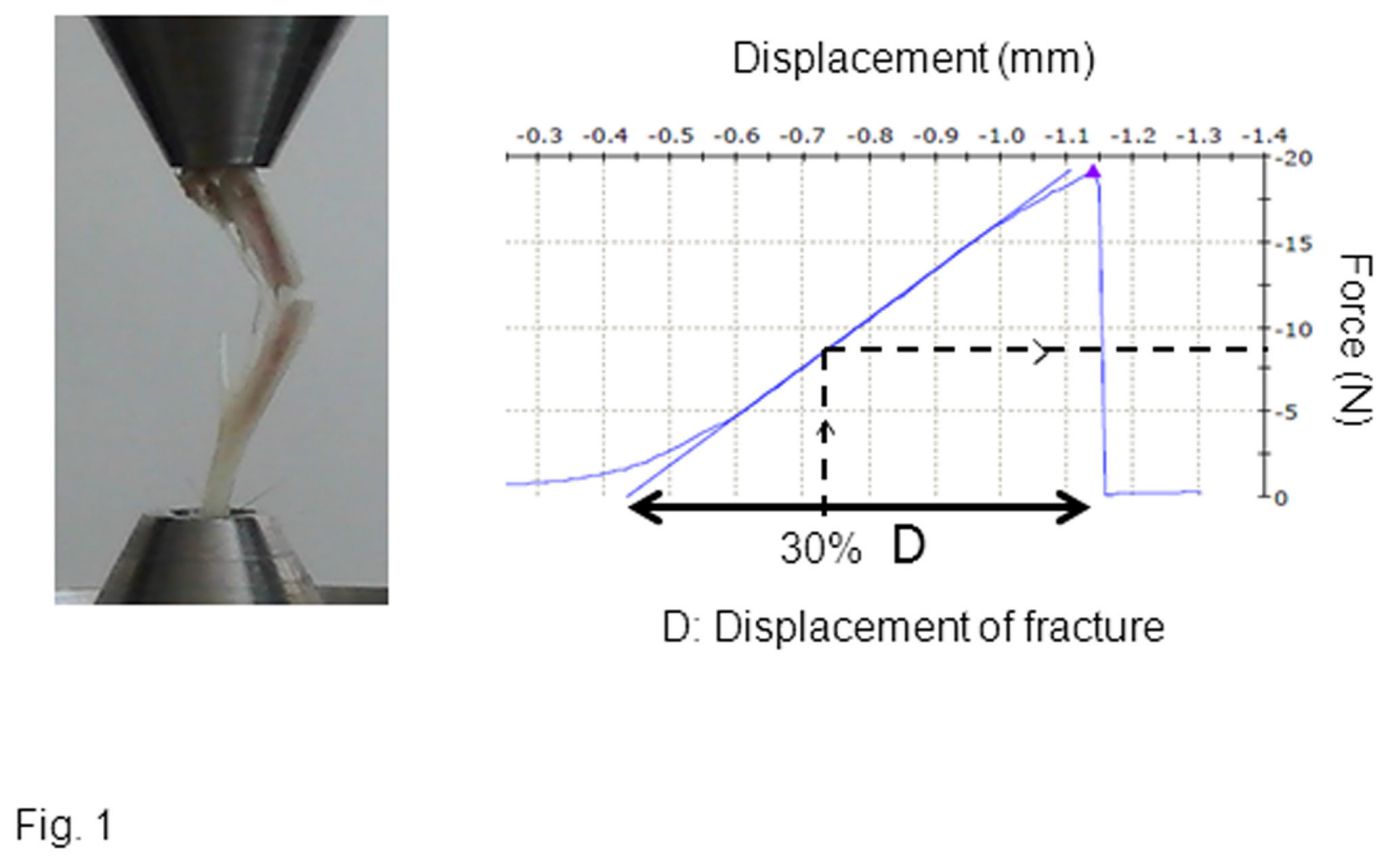
Axial compression of the full tibia. The mechanical resistance to failure was tested in pilot experiments to determine the loads which will be applied in vivo to induce damage [15] The force compression threshold for fatigue loading was determined as a displacement that reached 30% of the average displacement to complete fracture in *Postn* ^*+/+*^ mice (D).

The loading apparatus was specifically adapted for mice tibiae as previously described [19]. Fatigue loading force was terminated when the increase in actuator displacement reached 30% of the average displacement at complete fracture obtain on preliminary ex-vivo axial compression of the tibia (Figure 1). The force obtain at 30% of fracture displacement was not significantly different between *Postn* ^*+/+*^ and *Postn* ^*-/-*^ (11.97±1.7N vs 10.1±0.9N), therefore we applied the same peak load value in the two genotype during fatigue loading. In vivo, animals were anesthetized (1-3% isoflurane), the left tibia of each mouse was subjected to dynamic axial stimulation, using the following parameters: peak load = 12N ; peak strain (midshaft cortex) = 1500με ; pulse period (trapeze shaped pulse) = 0.1 s ; rest time between pulses = 0.33 s ; full cycle frequency (pulse + rest) = 3 Hz. A total of 360 cycles (^~^ 18min) were applied and animal returned to their cages. The non-stimulated right tibia served as an internal control. The mice were stimulated once and were sacrificed later at different time point. None of the mice showed signs of lameness or decreased activity levels after recovery of fatigue (n=6-8 mice/group).

### Ex vivo measurement of microarchitecture

Micro-computed tomography (microCT UCT40, Scanco Medical AG, Basserdorf Switzerland) was used to assess trabecular bone volume fraction in the proximal tibia, and cortical bone geometry at the midshaft tibial diaphysis as previously described [19]. Briefly, trabecular and cortical bone regions were evaluated using isotropic 12 μm voxels. 

For the trabecular region, to eliminate the primary spongious, we analyzed one hundred slices from the 50 slices under the proximal growth plate. 

Tibial cortical geometry was assessed using 50 continuous CT slides (600 μm) located at the tibial midshaft. Images were segmented using a fixed threshold approach. Morphometric variables were computed from binarized images using direct, three-dimensional techniques that do not rely on prior assumptions about the underlying structure[28]. For the trabecular bone regions, we assessed the bone volume fraction (BV/TV, %), trabecular thickness (TbTh, μm), trabecular number (TbN, mm^-1^), trabecular connectivity density (Tb Conn Density, mm^-3^) and structural model index (SMI). The structure model index was measured to determine the prevalence of plate-like or rod-like trabecular structures, where 0 represents “plates” and 3 “rods” [28]. For cortical bone at the tibial midshaft, we measured the cortical tissue volume (CtTV, mm^3^), bone volume (CtBV, mm^3^), the marrow volume (BMaV, mm^3^) and the average cortical width (CtTh, μm). 

### Ex vivo measurement of cortical porosity by high-resolution microCT

Micro-computed tomography (microCT UCT50, Scanco Medical AG, Basserdorf Switzerland) was used to assess cortical bone lacunae in the proximal tibia in the same region of interest than the trabecular structure as describe above. We analyzed 30 slices from 1.8mm under the proximal growth plate corresponding to region of tibia under the highest strain during axial compression. This region of interest was previously defined and located at postero-medial region in the 1/3 proximal tibia [19].

Lacunae distribution based on the thickness of the lacunae allowed analysing two types of lacunae. First, lacunae lower than 6um was consider to be “osteocyte lacunae” as previously define by Vatsa et al [29]. We assessed the lacunae volume fraction (LaV/TV, %), and the degree of anisotropy (DA). DA defines the magnitude of preferred orientation of lacunae. The higher the DA, the more the lacunae is aligned with the principal axis relative to the other axes [30]. Secondly, lacunae thickness higher than 12um was termed porosity and included blood vessel, micro-damage and real pore. We assessed the porosity volume fraction (PoV/TV, %), porosity thickness (PoTh, μm) and porosity number (PoN, mm^-1^). Those analyses have been performed only in animals sacrificed 15 days after fatigue.

### Bone formation indices by histomorphometry (calcein labelling)

 To measure dynamic indices of bone formation, mice received subcutaneous injections of calcein (25mg/kg, Sigma, Buchs, Switzerland) 9 and 2 days before euthanasia. Femur were embedded in methyl-methacrylate (merck, Switzerland), and 20-μm-thick transversal sections of the midshaft were cut with a saw (FinOcut, Metkon, Instruments LTD) than sanded to 10-μm-thick and mounted unstained for evaluation of fluorescence. Five-μm thick sagital sections were cut with a Leica Corp. Polycut E microtome (Leica Corp. Microsystems AG, Glattburg, Switzerland) and stained with modified Goldner’s trichrome, and histomorphometric measurements were performed on the secondary spongiosa of the proximal tibia metaphysis and on the endocortical and periosteal bone surfaces in the middle of the tibia, using a Leica Corp. Q image analyser at 40X magnification. All parameters were calculated and expressed according to standard formulas and nomenclatures [31] : mineral apposition rate (MAR, µm/day), single labeled perimeter (sLS/BPm, %), and double-labeled perimeter (dLS/BPm, %). Mineralizing perimeter per bone perimeter (MS/BPm, %) was calculated by adding dLS/BPm and one-half sLS/BPm. Bone formation rate (BFR/BPm, µm^3^/µm^2^/day) was calculated as the product of MS/BPm and MAR. 

### Microcracks measurement by histomorphometry (fuschin stainning)

Following dissection and prior to microCT, specimens dedicated for microcrack investigations were kept in 70% ethanol. After microCT, the tibia were completely dehydrated and stained with basic fuchsin according to the protocol of Burr et al [32].

Bone specimens were bulk-stained in 1% basic fuchsin in a graded series of ethanol (80%, 95% and 100%) under a vacuum for a total of 8 h before being embedded in methylmathacrylate. Microcracks were defined as linear structures with basic fuchsin staining within and around the cracks[33]. Unstained cracks were considered artefact associated with tissue processing. Cracks number per bone perimeter (CsNb, 1/mm) and cracks surface per bone surface (CsS, %) were evaluated using a Leica Corp. Q image analyser at 40X magnification. Since we only measure microcracks with a length higher than 10um, we will talk about cracks or damage rather than microcracks or microdamage. Quantification of diffuse damage was not enough reproducible in our hand to be shown in the manuscript.

### Immunohistochemistry of Periostin

The right and left tibiae were excised 3 days after fatigue loading, and subsequently fixed in 4% paraformaldehyde overnight at 4°C. They were then decalcified in 19% EDTA and 4% phosphate-buffered formalin for 3 weeks. The tibiae were then dehydrated in an ascending series of ethanol, cleared in Propar (Anatech LTD, Battle Creek, MI), and embedded in paraffin blocks. 8µm-thick sections were cut from the blocks at the tibia mid-shaft level using a RM2155 microtome (Leica, Germany) and mounted on Superfrost Plus slides (Fisher Scientific, Pittsburg, PA). After sections were incubated at 60 °C for 1h, deparaffinized in xylene, and rehydrated in a descending series of ethanol, the sections were stained according to the protocol of previously described [19,20]. The primary antibody (rabbit anti-Periostin) (Ed Krug, MUSC, Charleston, SC) was diluted in Antibody Diluent (DAKO, Carpinteria, CA) to a final concentration of 1:6000 and we used reagent of Vector Kit according to the manufacture’s directions (Vector Laboratories, Burlingame, CA).

### Testing of mechanical resistance

The night before mechanical testing, bones were thawed slowly at 7°C and then maintained at room temperature. Ex-vivo axial compression of the full tibia was performed on previous bone to determine parameters of in-vivo fatigue protocol. Tibia was placed as shown in Figure 1. The mechanical resistance to failure was tested using a servo-controlled electromechanical system (Instron 1114, Instron corp., High Wycombe, UK) with actuator displaced at 2mm/minute.

Tibias after fatigue were loaded to fracture in three-point bending ex-vivo to determine whole-bone mechanical properties. The length of the tibia (distance from intermalleolar to intercondylar region) was measured using callipers with an integrated electronic digital display and the midpoint of the shaft was determined. The tibia then was placed on the material testing machine on two supports separated by a distance of 9.9 mm and load was applied to the midpoint of the shaft (anteroposterior axis), thus creating a three-point bending test. Between each preparation step, the specimens were kept immersed in physiological solution. The mechanical resistance to failure was tested using a servo-controlled electromechanical system (Instron 1114, Instron corp., High Wycombe, UK) with actuator displaced at 2mm/minute. Both displacement and load were recorded. Ultimate force (maximal load, measured in Newtons [N]), stiffness (slope of the linear part of the curve, representing the elastic deformation, N/mm), and energy (surface under the curve, N*mm) were calculated. Ultimate stress (N/mm^2^) and Young’s modulus (MPa) were determined by the equations previously described by Turner and Burr [34]. Reproducibility was 3.3% for midshaft tibia. 

### Intrinsic mechanical properties by nanoindentation

Bone material level properties were tested using a nanoindentation technique at the midshaft cortex. Force, elasticity and tissue hardness of the bone were determined from a typical load displacement curve obtained during indentation of a pyramidal diamond indenter that is pressed into the bone sample. Loading was applied on hydrated bone tissue samples A nano-hardness tester (NHT, CSM Instruments, Peseux, Switzerland) was used as follows; force–displacement data of a pyramidal diamond indenter that is pressed into a material are recorded as previously described [35]. Briefly, the indenter tip is loaded at a given depth into the sample and the load is then held constant, leading to a creeping of the material below the tip. For the nanoindentation tests, tibia were thawed and embedded in polymethylmethacrylate and cut transversely at the midshaft. Samples were rehydrated following a standardized protocol in 16 h saline solution before testing. The mechanical tests included five indentations in the 1/3 external part of the cortex (nominated periosteal surface), five others in the 1/3 internal part of the cortex (nominated endocortical surface) and five indent in the 1/3 mid-cortex (nominated intra-cortical surface). Indents were set to a 900 nm depth with an approximate speed 76 mN/min for both loading and unloading. Full rehydration occurs at this distance necessary for the nanoindenter of less than 1 μm from the surface of the sample and is stable for up to a period of 60 h [35]. At maximum load, a 5 s holding period was applied. The limit of the maximal allowable thermal drift was set to 0.1 nm/s. All tests were performed by a technician blinded to the genotype of each group.

### Collagen crosslinking

Biochemical assays of immature (dihydroxylysinonorleucine DHLNL and hydroxylysinonorleucine HLNL) and mature (pyridinoline PYD and deoxypyridinoline DPD) enzymatic collagen crosslinks were performed as described previously Biochemical assays of cross-links were performed as described previously [36]. Briefly, the proximal and the distal metaphysis of tibia were removed with a diamond saw and then the diaphysis was powered in liquid nitrogen-cooled freezer mill (Spex Centriprep, Metuchen, USA), demineralised with daily changing of 0.5M EDTA in 0.05M Tris Buffer, pH 7.4 for 48 h at 4°C and extensively washed with deionized water. The demineralized bone residue was suspended in phosphate-buffered saline (0.15M NaCl in 20 mM sodium phosphate buffer, pH 7.4) and reduced with NaBH4 and hydrolyzed by 6M HCl at 110°C during 20h. Bone hydrolysates were pre-fractionned by Separation Phase Extraction Chromatography on Chromabond® Cross-links Columns (Macherey Nagel GmbH & Co.KG, Düren, Germany) to remove interfering molecules. Then collagen crosslinks separation was performed on a C18 Atlantis® T3 reversed-phase column with heptafluorobutyric acid as ion-pairing reagent in an acetonitrile-water mobile phase using an HPLC system equipped of with an Alliance 2695 separation module, and a Waters Micromass® ZQ™ Single Quadrupole Mass Spectrometer (Waters Corp. Milford, MA, USA). The amount of PYD, DPD, DHLNL and HLNL were expressed per molecule of collagen determined by hydroxyproline HPLC assay (Biorad, Munchen, Germany). 

### Data analysis

We first tested the effects of fatigue within groups (*Postn* ^*-/-*^ and *Postn* ^*+/+*^) by paired or unpaired t-tests. In the mechanical loading experiments, we compared fatigued and non-fatigued tibia in the same animal using a paired t-test. 

To compare the effect of genotype and the response to fatigue, we used a two way ANOVA. As appropriate, post hoc testing was performed using Fisher’s protected Least Squares Difference (PLSD). The p of interaction between the genotype and fatigue was only mentioned when it was found to be significant. Differences were considered significant at p < 0.05. Data are presented as mean ± SEM.

## Results

### Increased bone damage in periostin-deficient mice

**Figure 2:**
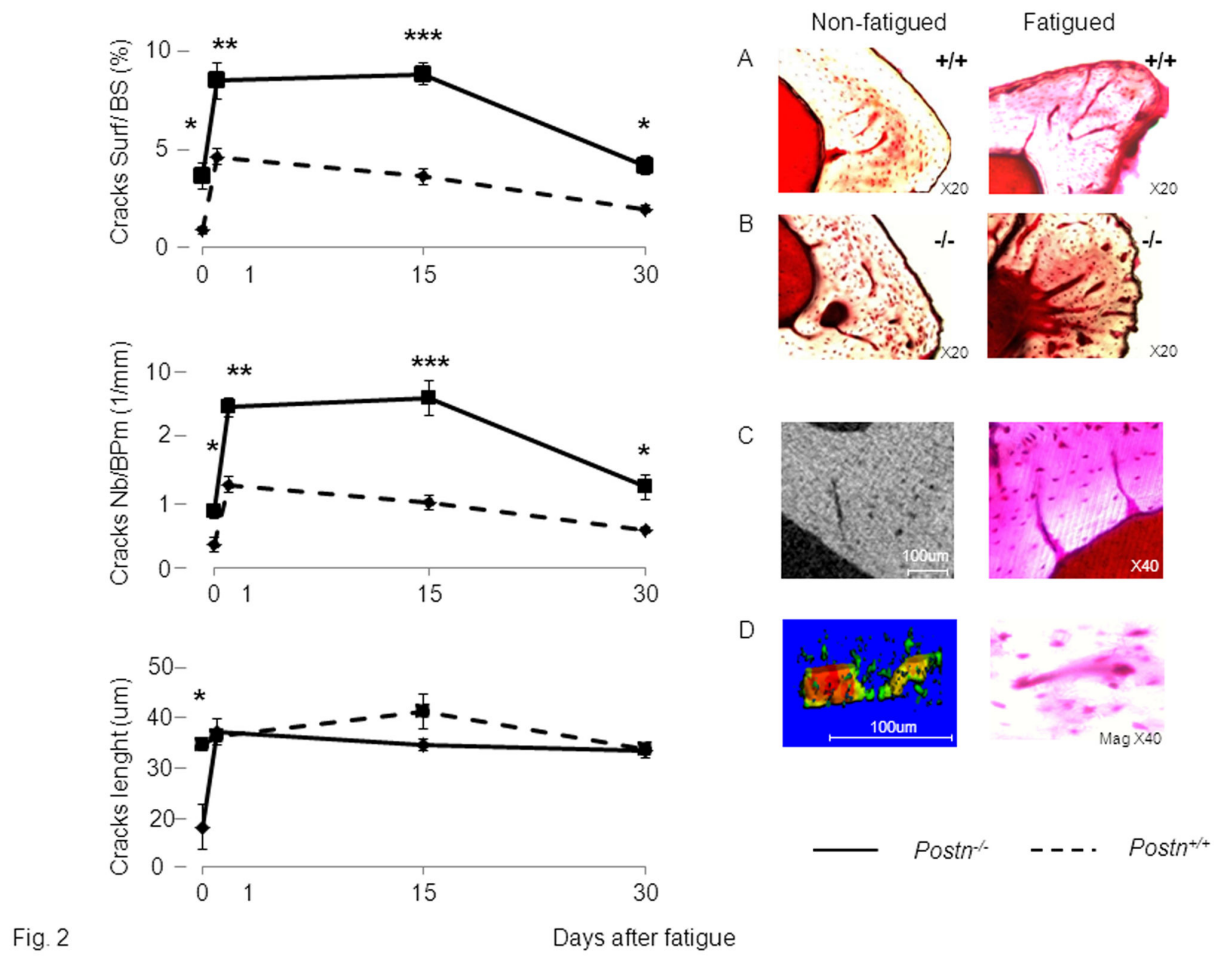
Effects of fatigue loading on bone damage in *Postn* ^*-/-*^ and *Postn* ^*+/+*^ mice. (A-B) Basic fuschin staining of cracks in *Postn* ^*+/+*^ and *Postn* ^*-/-*^ mice. (C) Cracks propagation visualized by 2D high resolution microCT (left panel), and confirmed by fuschin staining (right panel). (D) Cracks propagation from osteocyte lacunae to other osteocyte lacunae, visualized by 3D high resolution microCT (left panel), and confirmed by fuschin staining (right panel). * p<0.05,** p<0.01,*** p<0.001 indicate significant differences between fatigued tibia of *Postn* ^*-/-*^ vs *Postn* ^*+/+*^. Bars show mean (± sem), intermittent line: *Postn* ^*-/-*^, continuous line: *Postn* ^*+/+*^.

In *Postn* ^*+/+*^ mice, a marked increase in cracks number (CsNb, +252%) and surface (CsS, +433%) was already detectable 1 hour after the axial compression (p<0.001 vs non-fatigued tibia) (Figure 2). Fifteen days after fatigue, damage had started to decrease, but the trend was not yet significant (CsS, -21%, CsNb, -22% vs +1h). Thirty days after fatigue, however, the amount and extent of damage was significantly reduced (CsS, -58% , CsNb -54% vs +1h, p<0.01), although it remained higher compared to the non-fatigued tibia. Contrasting with *Postn* ^*+/+*^ mice, cracks were detectable in *Postn* ^*-/-*^ mice even in the absence of fatigue loading, i.e. in the non-fatigued tibia (CsS, +76%, CsNb +58%, respectively vs *Postn* ^*+/+*^, all p<0.05) (Figure 2). Axial compression increased damage in *Postn* ^*-/-*^ similarly to *Postn* ^*+/+*^mice, so the immediate post-fatigue level of damage remained higher in *Postn* ^*-/-*^ compared to *Postn* ^*+/+*^ mice (CsS, +83.8%, CsNb, +103%, p<0.01). Fifteen days after fatigue, damage levels were sustained in *Postn* ^*-/-*^, therefore the difference in cracks surface and number between *Postn* ^*-/-*^ and *Postn* ^*+/+*^ was further increased (CsS, +143%, CsNb, +147%, both p<0.001). Thirty days after fatigue, damage had eventually started to decline in *Postn* ^*-/-*^ (CsS, -50%, CsNb, -49% vs +1h, p<0.01), but still remained significantly higher compared to *Postn* ^*+/+*^ (Figure 2). 

**Figure 3:**
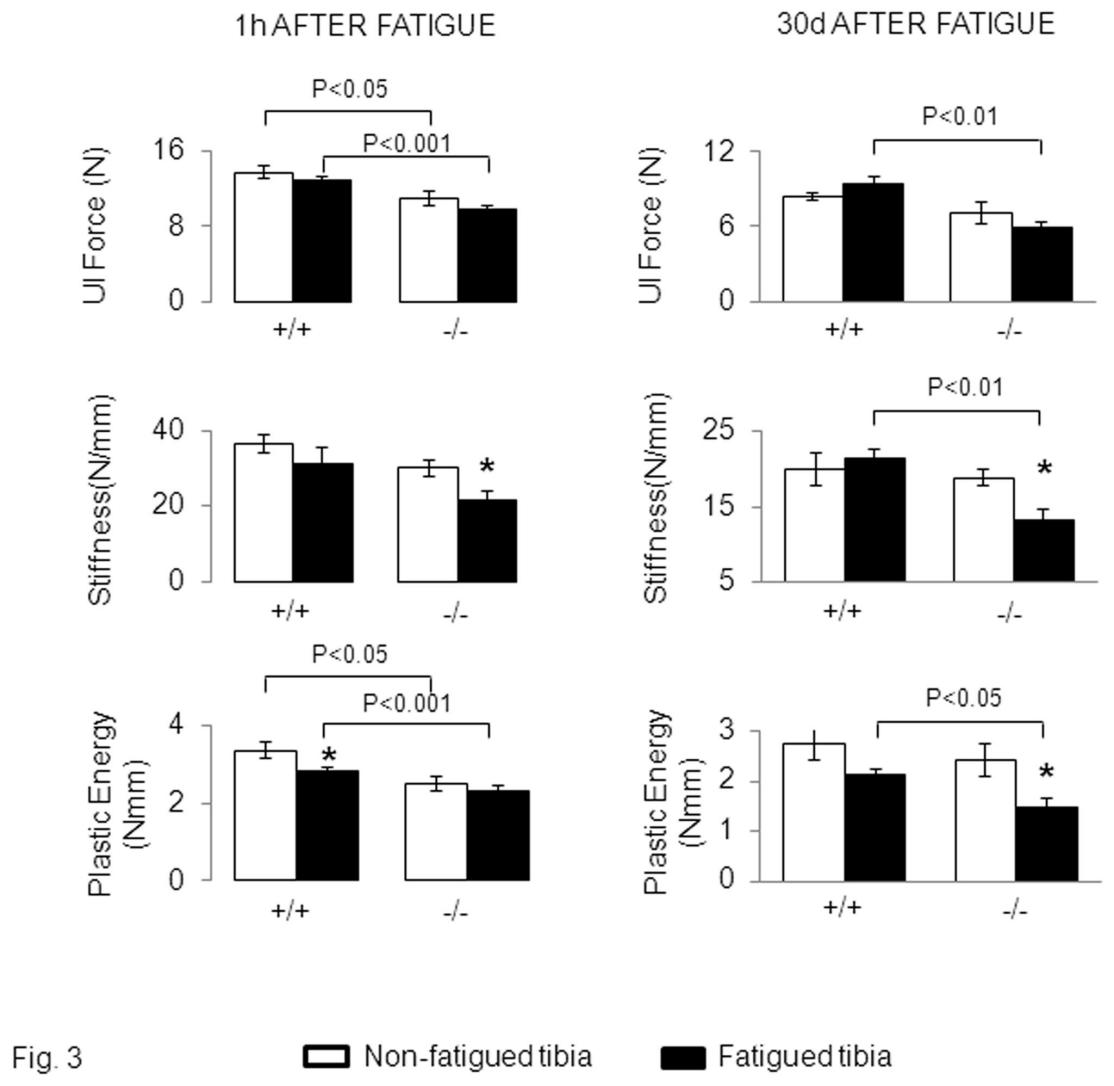
Effects of fatigue loading on bone mechanical properties in *Postn* ^*-/-*^ and *Postn* ^*+/+*^ mice. Biomechanical properties of the cortical tibia evaluated by three point bending, 1 hour and 30days after fatigue. Group differences were determined by post-hoc Fisher’s PLSD following 2F-ANOVA (fatigue-genotype). * p<0.05 compared to the non-fatigued tibia. Bars show mean (± sem) of n= 6-8 animals / group.

The amount of fatigue damage translated into significant differences in the bone biomechanical properties between *Postn* ^*+/+*^ and *Postn* ^*-/-*^ mice. In *Postn* ^*+/+*^, plastic energy was significantly decreased in the fatigued vs the non-fatigued tibia one hour after compression (Figure 3). Paralleling the progressive removal of damage in these mice, the plastic energy had recovered 30 days after fatigue. In contrast, in *Postn* ^*-/-*^, the plastic energy remained significantly lower in the fatigued tibia after 30 days, (-37% vs non-fatigued tibia, p<0.05 (Figure 3). In these mice, stiffness was also decreased at both 1 hour and 30 days after fatigue. These results suggested that *Postn* ^*-/-*^ are more susceptible (have a lower threshold) to cracks accumulation and/or have less effective remodelling mechanisms.

### Altered bone material level properties in periostin-deficient mice

**Figure 4:**
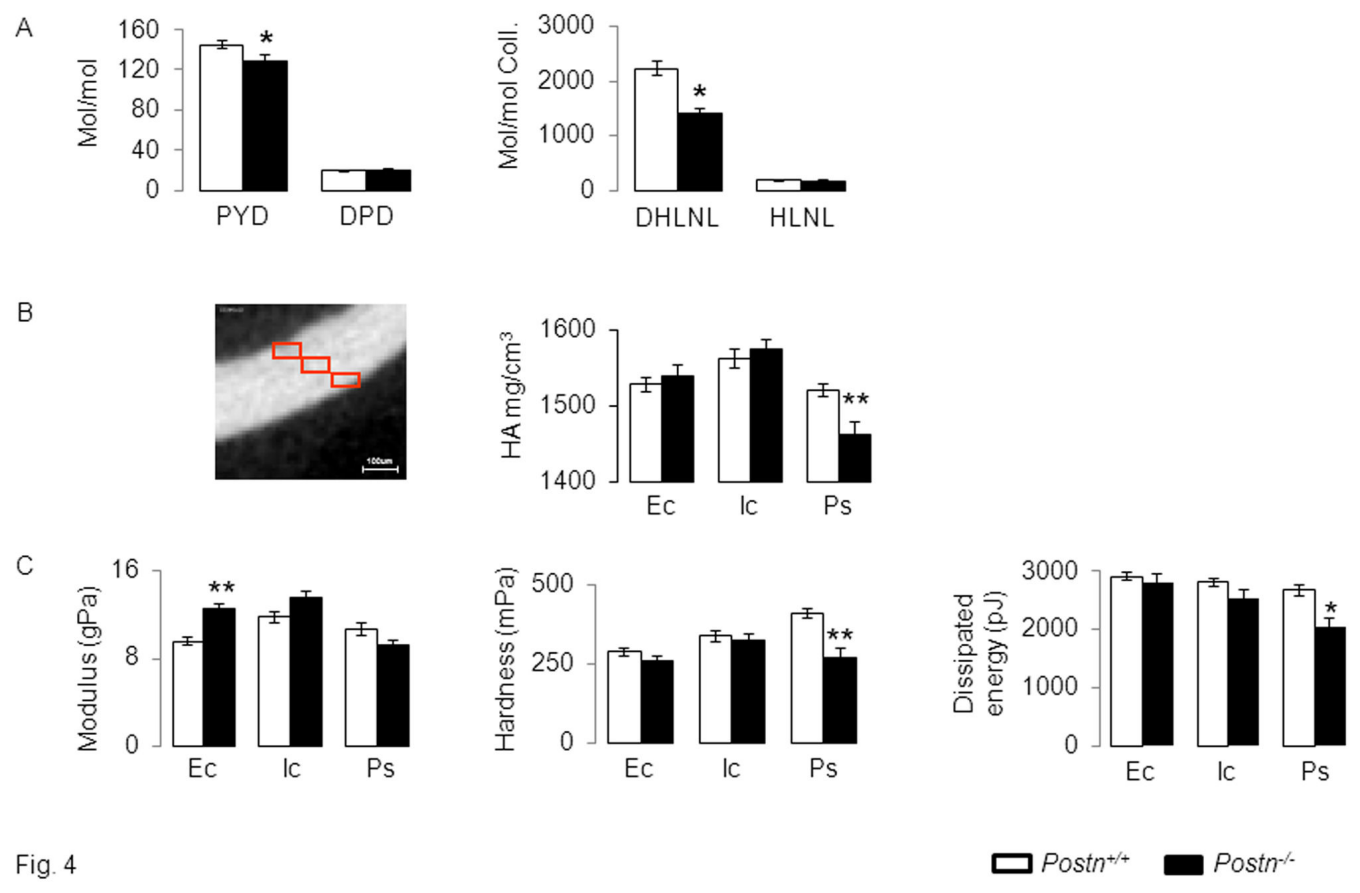
Collagen and bone material properties in *Postn* ^*-/-*^ and *Postn* ^*+/+*^ mice. (A) Concentration of immature (dihydroxylysinonorleucine, DHLNL and hydroxylysinonorleucine, HLNL) and mature (pyridinoline, PYD and deoxypyridinoline, DPD) crosslinks in bone extracts. (B) Volumetric BMD (HA: hydroxyapatite) evaluated by microCT in three identical cubes distributed from the outside to the inside of cortical bone (Ec, endocortical , Ic, intra-cortical, Ps, periosteal. (C) Bone material properties evaluated by nanoindentation. * p<0.05,** p<0.01 compared to *Postn* ^*+/+*^. Bars show mean (± sem) of n= 4 animals /gr.

Bone mass and microstructure is altered in *Postn* ^*-/-*^ mice[19,20]. We further evaluated the intrinsic bone quality parameters which could contribute to the higher damage levels in *Postn* ^*-/-*^ vs *Postn* ^*+/+*^. As shown in Figure 4, the bone content of both immature (DHLNL) and mature (PYD) enzymatic collagen crosslinks is lower in *Postn* ^*-/-*^ compared to *Postn* ^*+/+*^ mice. Moreover, the amount of DHLNL and PYD crosslinks was negatively associated with cracks surface (r2= 0.56, p=0.001 and r2=0.52, p=0.002, respectively).

Furthermore, the bone volumetric mineral density, modulus and hardness, respectively analyzed by microCT and nano-indentation at the 1/3^rd^ outer cortex, are lower in *Postn* ^*-/-*^ vs *Postn* ^*+/+*^mice. In contrast, the mineral density and material properties of trabeculae did not significantly differ between *Postn* ^*-/-*^ and *Postn* ^*+/+*^ mice (data not shown), consistent with the predominant expression of periostin in the cortical vs cancellous bone compartment [19]. 

### Lower intracortical remodelling in periostin-deficient mice

Next we investigated whether the largest difference in post-fatigue damage observed between *Postn* ^*-/-*^ and *Postn* ^*+/+*^mice, i.e. 15 days after loading, could be related to some indicators of intracortical bone remodelling. For this purpose, we first analyzed cortical “porosity” by microCT in the region of highest strain (see methods) [37]. 

**Figure 5:**
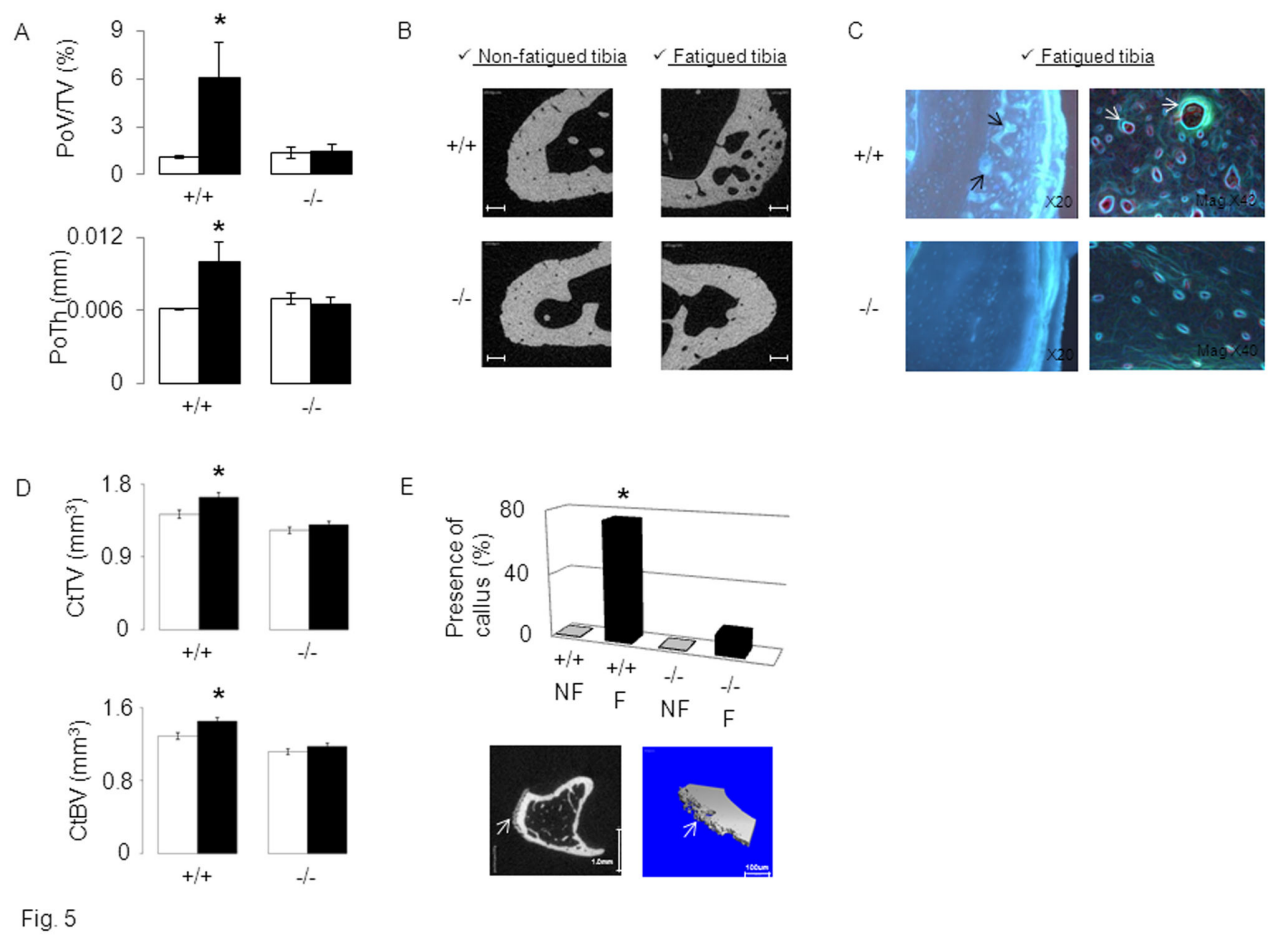
Post-fatigue intra-cortical remodeling and callus response in *Postn* ^*-/-*^ vs *Postn* ^*+/+*^ mice. Cortical bone parameters were evaluated by micro-CT 15 days after axial compression. A) Cortical porosity volume on tissue volume (PoV/TV) and Pore Thickness (PoTh). (B) 2D illustration of cortical porosity, bars scales 100um. (C) Intra-cortical remodeling indicated by calcein labeling around cortical pores (arrow indicate intense double labeling). (D) Cortical Bone Volume (CtBV) and Cortical Thickness (CtTh, mm) at proximal tibia. (E) Percent of mice presenting a callus in fatigued (F) vs non-fatigued (NF) tibia. Lower panel: arrow indicates periosteal woven bone on 2D and 3D micro-CT images of fatigued tibiae in *Postn* ^*+/+*^mice. * p<0.05 compared to non-fatigued tibia ( Fisher’s PLSD following 2F-ANOVA). Bars show mean (± sem) of n=6-8 animals /gr, black bars: fatigued tibia, white bars: non-fatigued tibia.

In the non-fatigued tibia, cortical porosity was extremely low and virtually not different between *Postn* ^*-/-*^ mice and *Postn* ^*+/+*^ littermates. However, PoV/TV, and PoTh were +452% and +64%, respectively, greater in the fatigued vs non-fatigued tibia of *Postn* ^*+/+*^mice (both p<0.05) (Figure 5). In contrast, in *Postn* ^*-/-*^ mice, and despite a greater absolute number of cracks (see above), porosity was not significantly increased in the fatigued compared to the non-fatigued bone. Histomorphometry confirmed the absence of intra-cortical remodelling in *Postn* ^*-/-*^ mice, whereas intense calcein labelling was observed in fatigued tibia of *Postn* ^*+/+*^ mice (Figure 5E). 

**Figure 6:**
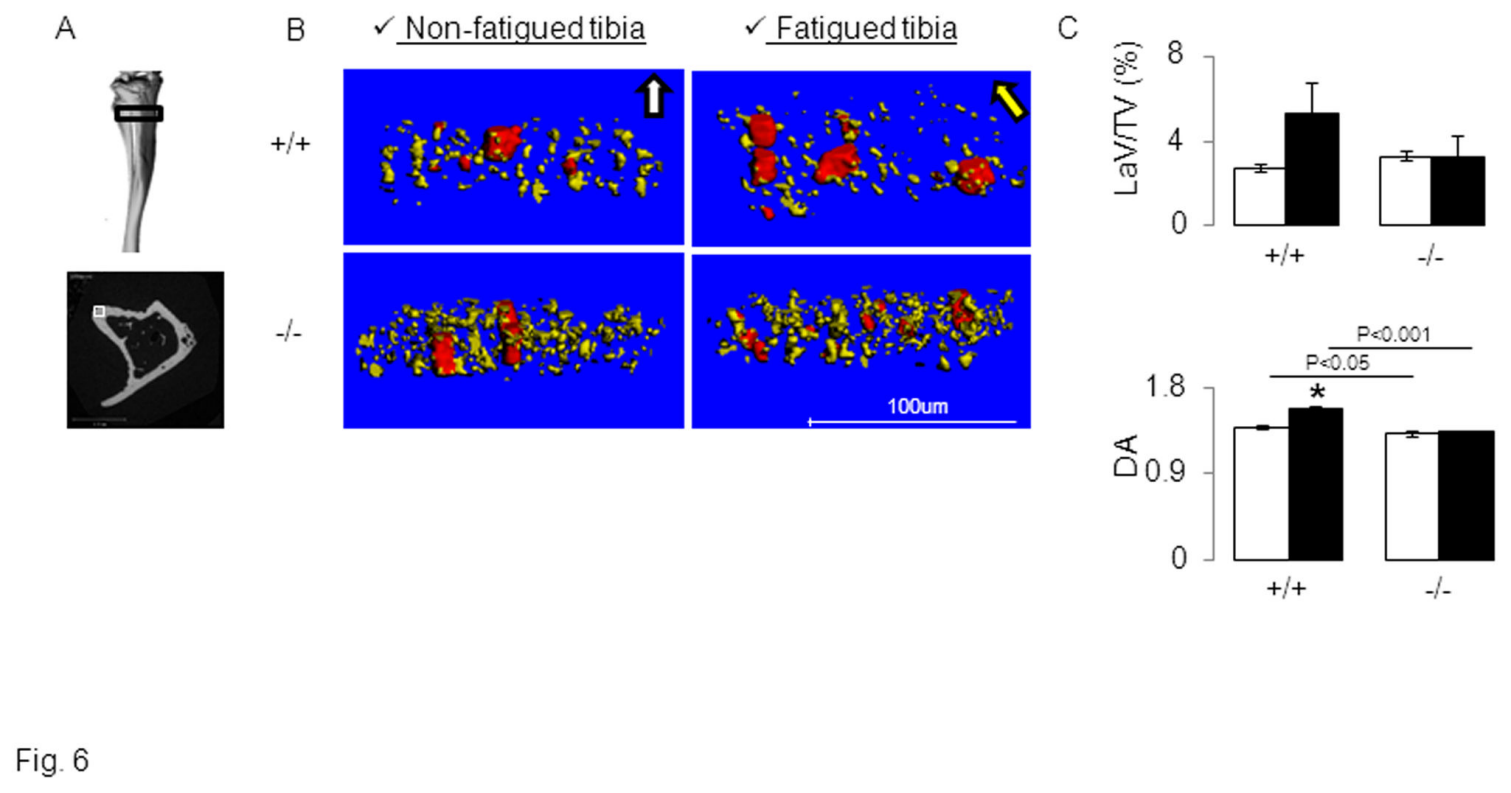
Effects of fatigue loading on the orientation of osteocyte lacunae in *Postn* ^*-/-*^ vs *Postn* ^*+/+*^ mice. 3D reconstructions of osteocytic lacunae were obtained by high resolution-computed tomography scans. (A) Upper panel: The black box illustrates the region of interest, 30 slices from 1.8mm under the proximal growth plate, in which a small cortical region located in the postero-lateral surface of the tibia (Lower panel, white box) was eventually selected for 3D reconstructions. (B) 3 D reconstructions of the canal network (red tubes corresponding to blood vessels or real pores) and osteocyte lacunae (yellow ellipsoids) show the alignment of osteocyte lacunae parallel to the principal direction of mechanical loading in tibia of *Postn* ^*+/+*^mice. In contrast, *Postn* ^*-/-*^ do not present oriented osteocyte lacunae either in the non-fatigued or fatigued tibia. (C) Lacunae volume on tissue volume (LaV/TV) and Degree of anisotropy (DA). * p<0.05 vs non-fatigued tibia. Bars show mean (± sem), black bars: fatigued tibia, white bars: non-fatigued tibia.

There is increasing evidence that osteocytes can participate to bone remodelling by regulating osteoclastogenesis[38], but also by a proper resorbing activity (osteocytic osteolysis) which translates into a reorganization of osteocytic lacunae. Hence osteocyte lacunae volume and orientation has been used as an index of osteocyte adaptation to loading[29]. The volume of osteocytic lacunae was not significantly different between *Postn* ^*-/-*^ and *Postn* ^*+/+*^ mice in absence of fatigue, but tended to increase in fatigued tibiae of *Postn* ^*+/+*^ mice (Figure 6). However the degree of anisotropy (DA) of osteocyte lacunae in the non-fatigued tibia was significantly lower in *Postn* ^*-/-*^ vs *Postn* ^*+/+*^ mice (Figure 6C), indicating that periostin contributes to the directional orientation of osteocytic lacunae. In *Postn* ^*+/+*^, fatigue further increased DA (+14.3% vs non-fatigued tibia, p<0.05), showing a higher orientation of the lacunae in the principal direction of the axial compression. In contrast in *Postn* ^*-/-*^, DA did not change in fatigued vs non-fatigued tibia (Figure 6B-C).

### Decreased callus formation in periostin-deficient mice

Then we investigated if periostin mediates the modeling injury response to fatigue loading by evaluating callus formation with both microCT and histology. One day after fatigue, no callus was detectable in *Postn* ^*+/+*^ nor *Postn* ^*-/-*^ mice. At day 7, in *Postn* ^*+/+*^, six out of eight of the fatigued tibia presented a callus, whereas a callus was seen in only one out of eight fatigued tibia from *Postn* ^*-/-*^ mice (Figure 5). As a consequence, in *Postn* ^*+/+*^ mice, fatigue significantly increased CtTV and CtBV (+14.6% and +12.7% vs non-fatigued tibia, respectively, p<0.05), whereas no significant changes in cortical microarchitecture were observed in *Postn* ^*-/-*^ mice. At days 15 and 30, the effects of fatigue on CtTV and CtBV were maintained in *Postn* ^*+/+*^ but still undetectable in *Postn* ^*-/-*^ mice. Fatigue did not significantly change trabecular structure either within *Postn* ^*+/+*^ or *Postn* ^*-/-*^ mice (Table 1).

**Table 1:** Influence of Postn on changes of tibial bone microarchitecture in response to fatigue.

		*Baseline*		*15 days after fatigue*		*30 days after fatigue*	
***NON-FATIGUED***		*Postn^+/+^*	*Postn^-/-^*	*Postn^+/+^*	*Postn^-/-^*	*Postn^+/+^*	*Postn^-/-^*
*Trabecular*	BV/TV (%)	8.6 ± 1.2	9.9 ± 1.8	10.2 ± 1.2	9.0 ± 0.7	11.6 ± 0.8	8.9 ± 1.1 *
	TbN (1/mm)	3.86 ± 0.18	4.40 ± 0.28	4.18 ± 0.12	3.94 ± 0.17	4.27 ± 0.12	3.88 ± 0.25
	TbTh (µm)	48.0 ± 2.1	44.6 ± 1.5	48.2 ± 1.4	47.4 ± 1.4	50.0 ± 2.6	48.3 ± 1.6
*Proximal-CorticalMidshaft-Cortical*	TV (mm^3^)	2.81 ± 0.08	2.50 ± 0.15***	3.31 ± 0.11	2.74 ± 0.10***	3.04 ± 0.10	2.60 ± 0.04***
	BV (mm^3^)	1.25 ± 0.04	0.93 ± 0.04**	1.47 ± 0.06	1.23 ± 0.04**	1.26 ± 0.04	1.05 ± 0.02**
	TV (mm^3^)	0.67 ± 0.02	0.54 ± 0.03**	0.76 ± 0.03	0.57 ± 0.02**	0.76 ± 0.05	0.58 ± 0.01**
	BV (mm^3^)	0.41 ± 0.01	0.33 ± 0.02**	0.46 ± 0.02	0.36 ± 0.01**	0.46 ± 0.02	0.36 ± 0.01**
***FATIGUED***							
*TrabecularProximal-Cortical*	BV/TV (%)	8.9 ± 1.1	9.8 ± 1.9	10.4 ± 1.1	9.6 ± 0.7	11.2 ± 0.9	8.6 ± 1.1
	TbN (1/mm)	3.84 ± 0.12	4.29 ± 0.45	4.25 ± 0.13	3.94 ± 0.17	4.35 ± 0.14	3.86 ± 0.26
	TbTh (µm)	48.5 ± 1.8	45.7 ± 1.5	49.9 ± 1.2	48.9 ± 1.3	48.0 ± 1.6	48.9 ± 1.0
	TV (mm^3^)	2.82 ± 0.10	2.44 ± 0.16	3.61 ± 0.14^$^	2.80 ± 0.11	3.25 ± 0.10 ^$^	2.77 ± 0.08 ^$^
*Midshaft-Cortical*	BV (mm^3^)	1.24 ± 0.05	0.95 ± 0.05	1.60 ± 0.07^$^	1.29 ± 0.05	1.33 ± 0.04	1.11 ± 0.03
	TV (mm^3^)	0.66 ± 0.03	0.54 ± 0.02	0.74 ± 0.03	0.58 ± 0.02	0.77 ± 0.04	0.58 ± 0.02
	BV (mm^3^)	0.40 ± 0.02	0.33 ± 0.02	0.45 ± 0.02	0.36 ± 0.01	0.47 ± 0.02	0.37 ± 0.01

Cancellous bone microarchitecture was evaluated at proximal tibia and cortical microarchitecture at midshaft tibia in the axially compressed bone and its controlateral non-stimulated control bone by in-vivo microCT at baseline, 15 days and 30 days after fatigue (n=8 mice / group). $ p<0.05 vs non-fatigued (paired t test). P value for differences between periostin groups independtly of fatigue, * p<0.05, ** p<0.01, *** p<0.001 Postn^-/-^ vs Postn^+/+^. Means ± SEM.

**Figure 7:**
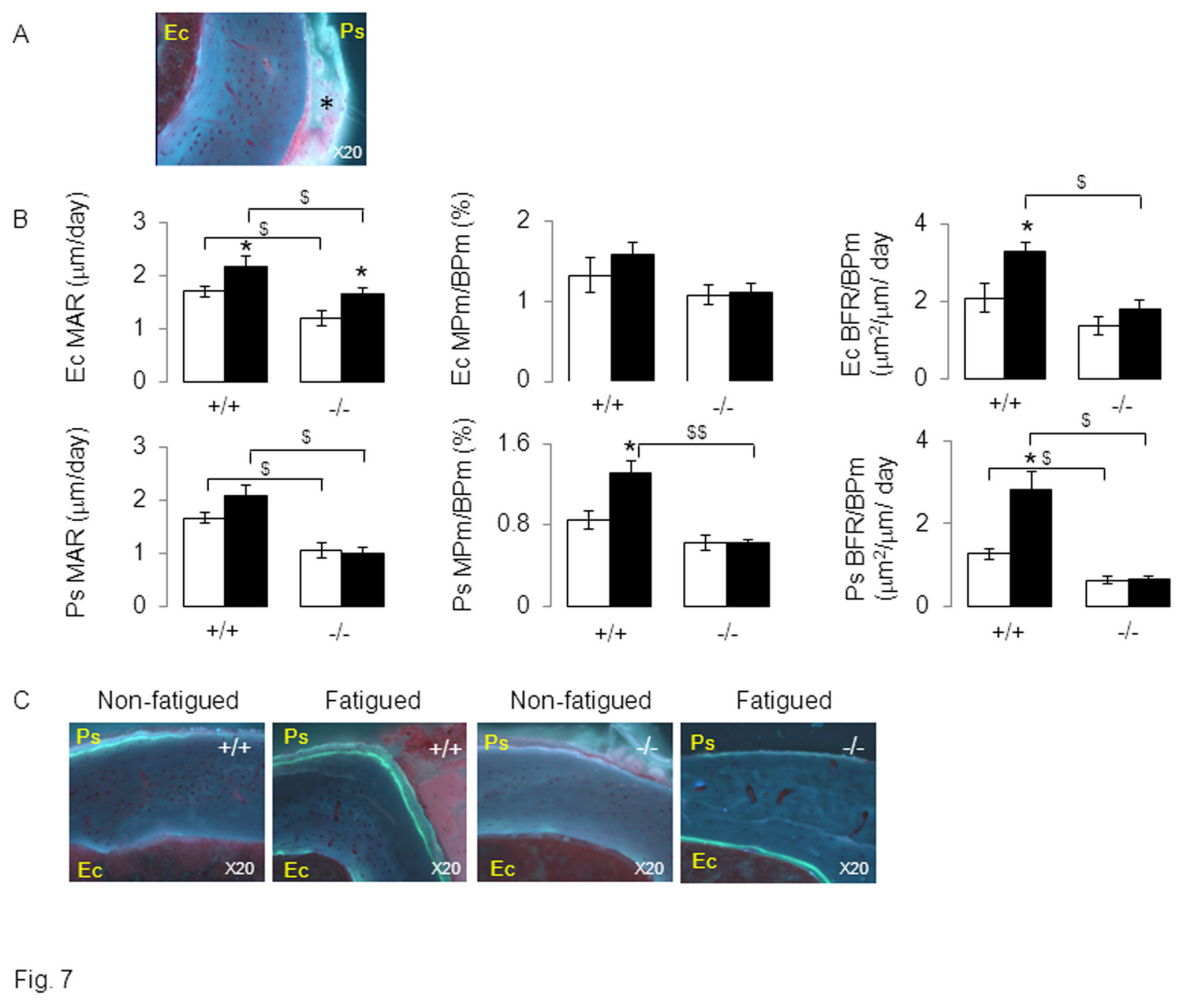
Effects of fatigue loading on bone formation indices in *Postn* ^*-/-*^ and *Postn* ^*+/+*^ mice. (A) Histological section of periosteal woven bone 15 days after fatigue. * indicate disorganized calcein labeling in the woven bone of *Postn* ^*+/+*^ mice. (B) Bone formation indices at endocortical (Ec) and periosteal (Ps) surfaces. (C) Fluorescent photomicrograph sections of midshaft tibia 30 days after fatigue showing measurable cortical calcein labels on periosteal and endosteal surfaces. Ps: periosteum, Ec: endocortical, mineral apposition rate (MAR), mineralisation perimeter (MPm), Bone formation rate (BFR), bone perimeter (BPm). * p<0.05 vs non-fatigued tibia. $ p<0.05 vs *Postn* ^*+/+*^. Bars show mean (± sem), black bars: fatigued tibia, white bars: non-fatigued tibia.

**Figure 8:**
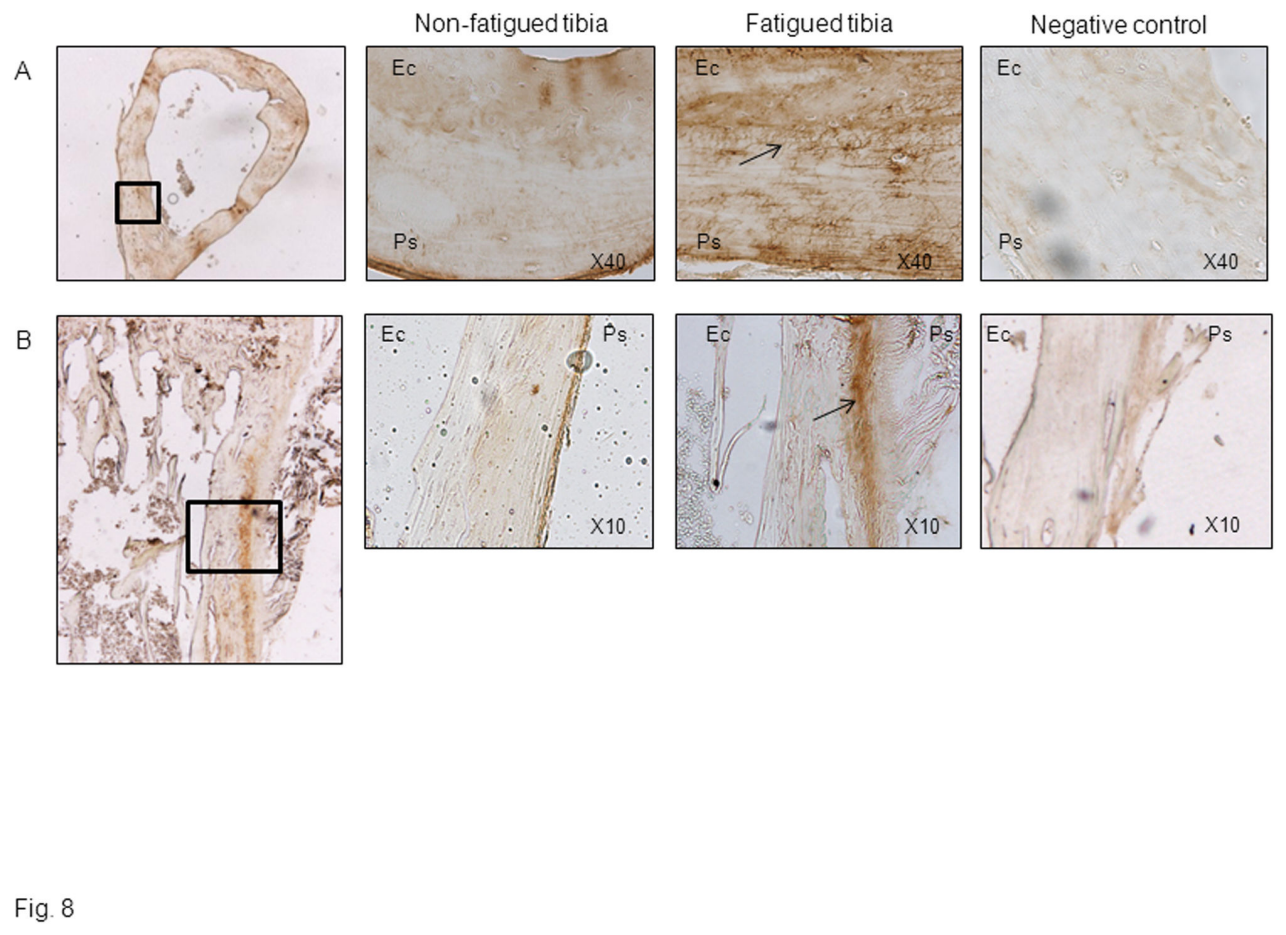
Fatigue loading stimulates periostin expression in cortical bone. Immunohistochemical staining of periostin expression in cross sections (A) and longitudinal sections (B) of the loaded and non-loaded midshaft tibia 3days after fatigue. Magnified images of the cortical region underlying soft calluses are also shown. Note the presence of periostin into osteocyte canaliculae of the fatigued tibia (A, arrow), as well as in the periosteum (Ps) of fatigued tibiae, whereas weak or no staining is detectable at endocortical surfaces (Ec).

Bone histomorphometry confirmed that *Postn* ^*+/+*^ mice had increased woven bone formation 30 days after fatigue loading (Figure 7). Hence, at the periosteum, fatigue increased the bone formation rate (BFR) +124% and the mineralisation perimeter (MPm/BPm) +55% compared to the non-fatigued tibia (p<0.05, Figure 7). Moreover, immunohistochemical staining of periostin in *Postn* ^*+/+*^ mice revealed a robust periosteal expression in the vicinity of the soft callus formation by day 3, as well as in osteocyte lacunae across the cortex (Figure 8). 

In contrast in *Postn* ^*-/-*^ mice, fatigue did not significantly increase periosteal MPm/BPm nor BFR. At endocortical surfaces, BFR also increased in response to fatigue in *Postn* ^*+/+*^ mice but not in Postn^-/-^ mice, confirming that both cortical modelling and remodelling was altered in periostin-deficient mice.

Since we previously reported that periostin is an important inhibitor of sclerostin expression [19,20], we eventually evaluated Sost mRNA levels in the fatigued tibiae of *Postn* ^*+/+*^ and *Postn* ^*-/-*^ mice. Sost increased more than 250% 1h after loading in *Postn* ^*+/+*^ mice, followed by a return towards baseline expression levels by 15 days after fatigue. Sost expression also increased 1hr post-loading in *Postn* ^*-/-*^ mice, but remained elevated (about 2 fold) up to 15 days, which could indeed contribute to the absence of callus formation in these mice.

## Discussion

The main objective of our study was to clarify the role of periostin in bone damage accumulation and repair in response to fatigue loading. Periostin deficiency alters bone mass and structure [19,20], and at the micron scale, mineralization and collagen organization [19,26]. As shown in this study, periostin deficiency further compromises collagen cross-linking and nanoindentation parameters such as hardness, potentially favouring the accumulation of cracks and reduction of bone strength. Furthermore, periostin-deficient mice not only fail to form new periosteal lamellar bone in response to physiological loading [19], but also to form woven bone (callus) in response to fatigue axial compression. Taken together with the evidence that axial compression increases Postn expression in the periosteum and osteocytes [19], and that Postn also increased in fatigued bones (data not shown), these observations confirm and expand the important role of periostin on bone modeling adaptive and injury responses [27]. They also suggest an influence of periostin on intra-cortical remodelling, as shown by the absence of cortical porosity and calcein labelling in *Postn* ^*-/-*^ mice, as well as by the absence of reorientation of osteocytes lacunae following fatigue loading in these mice [39]. 

Periostin deficiency significantly reduced the bone strength of fatigued tibia. This increased fragility likely reflects the combined effects of multiple mechanisms operating at different scales and at different time points. One day after fatigue, repair mechanisms (i.e. modeling and remodeling) did not have enough time to modify trabecular and/or cortical structure or other bone quality parameters (i.e crosslinks), so that the early increase in bone fragility is not due to microarchitecture or geometry changes [19]. At that time, the only parameters explaining changes in bone strength between non-fatigued and fatigued tibia are cracks accumulation. Our observations show an increase of cracks number and surface just after fatigue, and a significantly higher absolute amount of damage in fatigued tibia of *Postn* ^*-/-*^ vs *Postn* ^*+/+*^. In turn, the higher number of cracks in fatigued vs non-fatigued tibia of *Postn* ^*+/+*^ mice has significant repercussions on the plastic energy and only trends on the ultimate force. These results are clinically relevant, since damage accumulation with age has been associated more with a decreased of toughness (i.e amount of energy per volume that a material can absorb before rupturing) than with bone strength [40–42]. In contrast, in absence of periostin, cracks accumulation exerted a more prominent effect on stiffness. This indicates that hypomineralisation and lower collagen quantity / quality in *Postn* ^*-/-*^ [19,25,43], transfer the mechanical consequence of microcracks from toughness to stiffness, which to some degree is similar to the description made in osteogenesis imperfecta [44] or in MMP-13 deficient mice [45]. 

In wild-type, fifteen days after fatigue, cracks surface and number started to decrease and bone microarchitecture parameters were affected by callus formation and an increase of intracortical porosity. Unfortunately, at that time point, bone formation indices associated with the repair process were not measurable due to the disorganized structure of woven bone. Nevertheless, thirty days after fatigue, bone strength in the fatigued tibia of in *Postn* ^*+/+*^ mice had recovered, despite the fact that microcracks number and surface had not yet completely returned to their baseline values. This indicates that the periosteal injury response (callus) is enough to compensate for the remaining damage present in fatigued tibia. Periosteal woven bone formation has been shown to aid in the rapid recovery of whole bone strength, from 14 days to 35 days after fatigue depending on the severity of the fatigue protocol [7,15,33]. In absence of periostin though, cracks remained high and woven bone and porosity response to fatigue were inhibited. These observations indicate an absence of modeling and intra-cortical remodeling reaction in absence of periostin. To potentially explain the absence of modeling/remodeling two major hypotheses are suggested. First, absence of Postn altered the Sost response to fatigue loading, i.e. prevented its time-dependent decline after the initial increase. Consistent with this observation, we previously demonstrated that Sost inhibition by physiological loading also depends on Postn expression [19]. Second, periostin is also known to activate BMP and TGFβ through juxtacrine / paracrine and autocrine signaling [46]. BMP’s and TGFβ are known to be upregulated rapidly, almost 1h after fatigue loading, and their expression to remain elevated through 7 days [47,48], i.e. similarly to periostin. It is therefore possible that in bone cells periostin also contributes to BMP’s and TGFβ stimulation. 

Hence, fatigued tibia of periostin deficient mice still presented significant lower stiffness and plastic energy after 30 days. Altogether our results provide some evidence for a central role of periostin in the linear bone adaptive response to mechanical loading recently demonstrated between low and high strains with the lamellar/woven bone transition [49].

As previously shown, osteocyte lacunae adapt their morphology in response to loading [29], with more oriented lacunae in response to fatigue. The lack of reorientation and increased volume of osteocytic lacunae in *Postn* ^*-/-*^ could therefore suggest a potential role of periostin in the process of osteolytic osteolysis, usually observed in lactation model [50]. In others tissues, periostin expression has been suggested to represent an adaptive cell response to maintain cell survivals against an environmental stress [51,52]. Therefore, it is also possible that the higher rate of constitutive osteocytes apoptosis in *Postn* ^*-/-*^mice, which we previously demonstrated by staining of caspase 3 [20], interfered with the targeted remodeling process normally initiated by osteocytic death consecutive to microcracks [12]. 

There are several limitations to our study. First, the creation of cracks by a single bout of overloading in the order of 20 min does not replicate the time-course of the development of a clinical stress fracture. Most stress fractures develop over days, weeks or years, and are not caused solely by fatigue damage but by the interplay between fatigue damage, bone remodeling and local materials properties. More broadly, rodent models of bone fatigue are not perfect surrogates for the mechanisms of stress fractures in humans, since rodent bone is not osteonal in nature. A second limitation is that we applied the same amount of load to *Postn* ^*+/+*^ and *Postn* ^*-/-*^ mice despite the fact that they present initially different material and geometric properties. However, the increase in cracks number and surface immediately after loading was similar in *Postn* ^*+/+*^ and *Postn* ^*-/-*^, indicating that the displacement induced by the force applied during axial compression was not fundamentally different between these mice. Moreover, this point strengthen the demonstration of a major role of periostin in the injury response since it has been clearly demonstrated that the magnitude of the woven bone formation is scaled to the level of initial damage [15]. In absence of periostin, the absolute amount of damage was higher, which should have induced a magnified woven bone response, contrarily to our observation. Eventually, micro-CT evaluation of cortical porosity did not allow us to discriminate between true porosity, vascularization and damage. 

In summary, we have demonstrated that periostin-deficiency has significant consequences on bone materials properties, damage accumulation and repair, including local modeling/remodeling processes in response to fatigue. Whether changes in the dynamic levels of periostin expression in response to strenuous exercise, aging and/or anti-resorptives drugs do occur and could thereby contribute directly to the development of stress and atypical fractures remains to be investigated.

## References

[1] Burr DB, Forwood MR, Fyhrie DP, Martin RB, Schaffler MB et al. (1997) Bone microdamage and skeletal fragility in osteoporotic and stress fractures. J Bone Miner Res 12: 6-15. doi:10.1359/jbmr.1997.12.1.6. PubMed: 9240720. 9240720

[2] Schaffler MB, Choi K, Milgrom C (1995) Aging and matrix microdamage accumulation in human compact bone. Bone 17: 521-525. doi:10.1016/8756-3282(95)90512-X. PubMed: 8835305. 8835305

[3] Mori S, Harruff R, Ambrosius W, Burr DB (1997) Trabecular bone volume and microdamage accumulation in the femoral heads of women with and without femoral neck fractures. Bone 21: 521-526. doi:10.1016/S8756-3282(97)00200-7. PubMed: 9430242. 9430242

[4] Norman TL, Little TM, Yeni YN (2008) Age-related changes in porosity and mineralization and in-service damage accumulation. J Biomech 41: 2868-2873. doi:10.1016/j.jbiomech.2008.06.032. PubMed: 18703196. 18703196

[5] Burr DB, Turner CH, Naick P, Forwood MR, Ambrosius W et al. (1998) Does microdamage accumulation affect the mechanical properties of bone? J Biomech 31: 337-345. doi:10.1016/S0021-9290(98)00016-5. PubMed: 9672087. 9672087

[6] Danova NA, Colopy SA, Radtke CL, Kalscheur VL, Markel MD et al. (2003) Degradation of bone structural properties by accumulation and coalescence of microcracks. Bone 33: 197-205. doi:10.1016/S8756-3282(03)00155-8. PubMed: 14499353. 14499353

[7] Hsieh YF, Silva MJ (2002) In vivo fatigue loading of the rat ulna induces both bone formation and resorption and leads to time-related changes in bone mechanical properties and density. J Orthop Res 20: 764-771. doi:10.1016/S0736-0266(01)00161-9. PubMed: 12168665. 12168665

[8] Zioupos P (2001) Accumulation of in-vivo fatigue microdamage and its relation to biomechanical properties in ageing human cortical bone. J Microsc 201: 270-278. doi:10.1046/j.1365-2818.2001.00783.x. PubMed: 1143014011207929. 11207929

[9] Taylor D, Hazenberg JG, Lee TC (2007) Living with cracks: damage and repair in human bone. Nat Mater 6: 263-268. doi:10.1038/nmat1866. PubMed: 17401419. 17401419

[10] Burr DB, Martin RB, Schaffler MB, Radin EL (1985) Bone remodeling in response to in vivo fatigue microdamage. J Biomech 18: 189-200. doi:10.1016/0021-9290(85)90204-0. PubMed: 3997903. 3997903

[11] Hazenberg JG, Freeley M, Foran E, Lee TC, Taylor D (2006) Microdamage: a cell transducing mechanism based on ruptured osteocyte processes. J Biomech 39: 2096-2103. doi:10.1016/j.jbiomech.2005.06.006. PubMed: 16112124. 16112124

[12] Cardoso L, Herman BC, Verborgt O, Laudier D, Majeska RJ et al. (2009) Osteocyte apoptosis controls activation of intracortical resorption in response to bone fatigue. J Bone Miner Res 24: 597-605. doi:10.1359/jbmr.081210. PubMed: 19049324. 19049324 PMC2659511

[13] Xiong J, Onal M, Jilka RL, Weinstein RS, Manolagas SC et al. (2011 ) Matrix-embedded cells control osteoclast formation. Nat Med 17: 1235-1241. doi:10.1038/nm.2448. PubMed: 21909103. 21909103 PMC3192296

[14] Kennedy OD, Herman BC, Laudier DM, Majeska RJ, Sun HB et al. (2012) Activation of resorption in fatigue-loaded bone involves both apoptosis and active pro-osteoclastogenic signaling by distinct osteocyte populations. Bone 50: 1115-1122. doi:10.1016/j.bone.2012.01.025. PubMed: 22342796. 22342796 PMC3366436

[15] Uthgenannt BA, Kramer MH, Hwu JA, Wopenka B, Silva MJ (2007) Skeletal self-repair: Stress fracture healing by rapid formation and densification of woven bone. J Bone Miner Res 22: 1548-1556. doi:10.1359/jbmr.0070614. PubMed: 17576168. 17576168 PMC3680519

[16] Martin RB (2001) The role of bone remodeling in preventing or promoting stress fractures. In: Burr DB Milgrom C Musculoskeletal fatigue and stress fractures. Boca Raton: CRC Press pp 183-201.

[17] Rhee Y, Allen MR, Condon K, Lezcano V, Ronda AC et al. (2011) PTH receptor signaling in osteocytes governs periosteal bone formation and intracortical remodeling. J Bone Miner Res 26: 1035-1046. doi:10.1002/jbmr.304. PubMed: 21140374. 21140374 PMC3179307

[18] Li X, Ominsky MS, Warmington KS, Morony S, Gong J et al. (2009) Sclerostin antibody treatment increases bone formation, bone mass, and bone strength in a rat model of postmenopausal osteoporosis. J Bone Miner Res 24: 578-588. doi:10.1359/jbmr.081206. PubMed: 19049336. 19049336

[19] Bonnet N, Standley KN, Bianchi EN, Stadelmann V, Foti M et al. (2009) The matricellular protein Periostin is required for Sclerostin inhibition and the anabolic response to mechanical loading and physical activity. J Biol Chem 284: 35939-35950. doi:10.1074/jbc.M109.060335. PubMed: 19837663. 19837663 PMC2791022

[20] Bonnet N, Conway SJ, Ferrari SL (2012) Regulation of beta catenin signaling and parathyroid hormone anabolic effects in bone by the matricellular protein periostin. Proc Natl Acad Sci U S A 109: 15048-15053. doi:10.1073/pnas.1203085109. PubMed: 22927401. 22927401 PMC3443161

[21] Kühn B, del Monte F, Hajjar RJ, Chang YS, Lebeche D et al. (2007) Periostin induces proliferation of differentiated cardiomyocytes and promotes cardiac repair. Nat Med 13: 962-969. doi:10.1038/nm1619. PubMed: 17632525. 17632525

[22] Nakazawa T, Nakajima A, Seki N, Okawa A, Kato M et al. (2004) Gene expression of periostin in the early stage of fracture healing detected by cDNA microarray analysis. J Orthop Res 22: 520-525. doi:10.1016/j.orthres.2003.10.007. PubMed: 15099630. 15099630

[23] Gillan L, Matei D, Fishman DA, Gerbin CS, Karlan BY et al. (2002) Periostin secreted by epithelial ovarian carcinoma is a ligand for alpha(V)beta(3) and alpha(V)beta(5) integrins and promotes cell motility. Cancer Res 62: 5358-5364. PubMed: 12235007. 12235007

[24] Shimazaki M, Nakamura K, Kii I, Kashima T, Amizuka N et al. (2008) Periostin is essential for cardiac healing after acute myocardial infarction. J Exp Med 205: 295-303. doi:10.1084/jem.20071297. PubMed: 18208976. 18208976 PMC2271007

[25] Kii I, Nishiyama T, Li M, Matsumoto K, Saito M et al. (2010) Incorporation of tenascin-C into the extracellular matrix by periostin underlies an extracellular meshwork architecture. J Biol Chem 285: 2028-2039. doi:10.1074/jbc.M109.051961. PubMed: 19887451. 19887451 PMC2804360

[26] Merle B, Garnero P (2012) The multiple facets of periostin in bone metabolism. Osteoporos Int 23: 1199-1212. doi:10.1007/s00198-011-1892-7. PubMed: 22310955. 22310955

[27] Rios H, Koushik SV, Wang H, Wang J, Zhou HM et al. (2005) periostin null mice exhibit dwarfism, incisor enamel defects, and an early-onset periodontal disease-like phenotype. Mol Cell Biol 25: 11131-11144. doi:10.1128/MCB.25.24.11131-11144.2005. PubMed: 16314533. 16314533 PMC1316984

[28] Hildebrand T, Ruegsegger P (1997) A new method for the model-independent assessment of thickness in the three-dimensional images. J Microsc 185: 67-75. doi:10.1046/j.1365-2818.1997.1340694.x.

[29] Vatsa A, Breuls RG, Semeins CM, Salmon PL, Smit TH et al. (2008) Osteocyte morphology in fibula and calvaria - is there a role for mechanosensing? Bone 43: 452-458. doi:10.1016/j.bone.2008.01.030. PubMed: 18625577. 18625577

[30] Schneider P, Stauber M, Voide R, Stampanoni M, Donahue LR et al. (2007) Ultrastructural properties in cortical bone vary greatly in two inbred strains of mice as assessed by synchrotron light based micro- and nano-CT. J Bone Miner Res 22: 1557-1570. doi:10.1359/jbmr.070703. PubMed: 17605631. 17605631

[31] Parfitt AM, Drezner MK, Glorieux FH, Kanis JA, Malluche H et al. (1987) Bone histomorphometry: standardization of nomenclature, symbols, and units. Report of the ASBMR Histomorphometry Nomenclature Committee. J Bone Miner Res 2: 595-610. PubMed: 3455637. 3455637 10.1002/jbmr.5650020617

[32] Burr DB, Hooser M (1995 ) Alterations to the en bloc basic fuchsin staining protocol for the demonstration of microdamage produced in vivo. Bone 17: 431-433. doi:10.1016/S8756-3282(95)00241-3. PubMed: 8573418. 8573418

[33] Waldorff EI, Christenson KB, Cooney LA, Goldstein SA (2010) Microdamage repair and remodeling requires mechanical loading. J Bone Miner Res 25: 734-745. PubMed: 19821772. 19821772 10.1359/jbmr.091016 PMC3153328

[34] Turner CH, Burr DB (1993) Basic biomechanical measurements of bone: a tutorial. Bone 14: 595-608. doi:10.1016/8756-3282(93)90081-K. PubMed: 8274302. 8274302

[35] Brennan-Speranza TC, Rizzoli R, Kream BE, Rosen C, Ammann P (2011) Selective osteoblast overexpression of IGF-I in mice prevents low protein-induced deterioration of bone strength and material level properties. Bone 49: 1073-1079. doi:10.1016/j.bone.2011.07.039. PubMed: 21840432. 21840432

[36] Gineyts E, Borel O, Chapurlat R, Garnero P (2010) Quantification of immature and mature collagen crosslinks by liquid chromatography-electrospray ionization mass spectrometry in connective tissues. J Chromatogr B Anal Technol Biomed Life Sci 878: 1449-1454. doi:10.1016/j.jchromb.2010.03.039. PubMed: 20417158. 20417158

[37] Li CY, Jee WS, Chen JL, Mo A, Setterberg RB et al. (2003) Estrogen and "exercise" have a synergistic effect in preventing bone loss in the lumbar vertebra and femoral neck of the ovariectomized rat. Calcif Tissue Int 72: 42-49. doi:10.1007/s00223-001-1086-y. PubMed: 12370795. 12370795

[38] Nakashima T, Hayashi M, Fukunaga T, Kurata K, Oh-Hora M et al. (2011) Evidence for osteocyte regulation of bone homeostasis through RANKL expression. Nat Med 17: 1231-1234. doi:10.1038/nm.2452. PubMed: 21909105. 21909105

[39] Lotinun S, Kiviranta R, Matsubara T, Alzate JA, Neff L et al. (2013) Osteoclast-specific cathepsin K deletion stimulates S1P-dependent bone formation. J Clin Invest 123: 666-681. PubMed: 23321671. 23321671 10.1172/JCI64840 PMC3561821

[40] Currey JD, Brear K, Zioupos P (1996) The effects of ageing and changes in mineral content in degrading the toughness of human femora. J Biomech 29: 257-260. doi:10.1016/0021-9290(95)00048-8. PubMed: 8849821. 8849821

[41] Zioupos P, Currey JD (1998) Changes in the stiffness, strength, and toughness of human cortical bone with age. Bone 22: 57-66. doi:10.1016/S8756-3282(97)00228-7. PubMed: 9437514. 9437514

[42] Burr DB (2002) Bone material properties and mineral matrix contributions to fracture risk or age in women and men. J Musculoskelet Neuronal Interact 2: 201-204. PubMed: 15758433. 15758433

[43] Litvin J, Selim AH, Montgomery MO, Lehmann K, Rico MC et al. (2004 ) Expression and function of periostin-isoforms in bone. J Cell Biochem 92: 1044-1061. doi:10.1002/jcb.20115. PubMed: 15258926. 15258926

[44] Kozloff KM, Carden A, Bergwitz C, Forlino A, Uveges TE et al. (2004) Brittle IV mouse model for osteogenesis imperfecta IV demonstrates postpubertal adaptations to improve whole bone strength. J Bone Miner Res 19: 614-622. doi:10.1359/JBMR.040111. PubMed: 15005849. 15005849

[45] Tang SY, Herber RP, Ho SP, Alliston T (2012) Matrix metalloproteinase-13 is required for osteocytic perilacunar remodeling and maintains bone fracture resistance. J Bone Miner Res 27: 1936-1950. doi:10.1002/jbmr.1646. PubMed: 22549931. 22549931 PMC3415585

[46] Conway SJ, Doetschman T, Azhar M (2011) The inter-relationship of periostin, TGF beta, and BMP in heart valve development and valvular heart diseases. ScientificWorldJournal 11: 1509-1524. doi:10.1100/tsw.2011.132. PubMed: 21805020. 21805020 PMC5548286

[47] Kidd LJ, Stephens AS, Kuliwaba JS, Fazzalari NL, Wu AC et al. (2010) Temporal pattern of gene expression and histology of stress fracture healing. Bone 46: 369-378. doi:10.1016/j.bone.2009.10.009. PubMed: 19836476. 19836476

[48] Wohl GR, Towler DA, Silva MJ (2009) Stress fracture healing: fatigue loading of the rat ulna induces upregulation in expression of osteogenic and angiogenic genes that mimic the intramembranous portion of fracture repair. Bone 44: 320-330. doi:10.1016/j.bone.2009.03.605. PubMed: 18950737. 18950737 PMC2759644

[49] Sugiyama T, Meakin LB, Browne WJ, Galea GL, Price JS et al. (2012) Bones' adaptive response to mechanical loading is essentially linear between the low strains associated with disuse and the high strains associated with the lamellar/woven bone transition. J Bone Miner Res 27: 1784-1793. doi:10.1002/jbmr.1599. PubMed: 22431329. 22431329 PMC3427886

[50] Qing H, Ardeshirpour L, Pajevic PD, Dusevich V, Jähn K et al. (2012) Demonstration of osteocytic perilacunar/canalicular remodeling in mice during lactation. J Bone Miner Res 27: 1018-1029. doi:10.1002/jbmr.1567. PubMed: 22308018. 22308018 PMC3770147

[51] Rios HF, Ma D, Xie Y, Giannobile WV, Bonewald LF et al. (2008) Periostin Is Essential for the Integrity and Function of the Periodontal Ligament During Occlusal Loading in Mice. J Periodontol 79: 1480-1490. doi:10.1902/jop.2008.070624. PubMed: 18672999. 18672999 PMC2581516

[52] Ma D, Lu H, Xu L, Xu X, Xiao W (2009) Mechanical loading promotes Lewis lung cancer cell growth through periostin. In Vitro Cell Dev Biol Anim 45: 467-472. doi:10.1007/s11626-009-9214-5. PubMed: 19533256. 19533256

